# Investigation of the Potential of Selected Food-Derived Antioxidants to Bind and Stabilise the Bioactive Blue Protein C-Phycocyanin from Cyanobacteria Spirulina

**DOI:** 10.3390/ijms25010229

**Published:** 2023-12-22

**Authors:** Nikola Gligorijević, Zorana Jovanović, Ilija Cvijetić, Miloš Šunderić, Luka Veličković, Jaroslav Katrlík, Alena Holazová, Milan Nikolić, Simeon Minić

**Affiliations:** 1 University of Belgrade, Institute of Chemistry, Technology and Metallurgy, Department of Chemistry, National Institute of the Republic of Serbia, Studentski trg 12–16, 11000 Belgrade, Serbia; nikola.gligorijevic@ihtm.bg.ac.rs; 2 University of Belgrade-Faculty of Chemistry, Center of Excellence for Molecular Food Sciences & Department of Biochemistry, Studentski trg 12–16, 11000 Belgrade, Serbia; zorana.gr@gmail.com (Z.J.); luka@chem.bg.ac.rs (L.V.); 3 University of Belgrade-Faculty of Chemistry, Department of Analytical Chemistry, Studentski trg 12–16, 11000 Belgrade, Serbia; ilija@chem.bg.ac.rs; 4 University of Belgrade-Institute for the Application of Nuclear Energy (INEP), Banatska 31b, 11000 Belgrade, Serbia; milos@inep.co.rs; 5 Institute of Chemistry, Slovak Academy of Sciences, Dúbravská cesta 5807/9, 84538 Bratislava, Slovakia; katrlik@yahoo.com (J.K.); alenasediva15@gmail.com (A.H.)

**Keywords:** C-phycocyanin, spirulina, food antioxidants, quercetin, resveratrol, coenzyme Q_10_, binding interactions, spectroscopic methods, molecular docking, protein stabilisation

## Abstract

Blue C-phycocyanin (C-PC), the major Spirulina protein with innumerable health-promoting benefits, is an attractive colourant and food supplement. A crucial obstacle to its more extensive use is its relatively low stability. This study aimed to screen various food-derived ligands for their ability to bind and stabilise C-PC, utilising spectroscopic techniques and molecular docking. Among twelve examined ligands, the protein fluorescence quenching revealed that only quercetin, coenzyme Q_10_ and resveratrol had a moderate affinity to C-PC (*K*_a_ of 2.2 to 3.7 × 10^5^ M^–1^). Docking revealed these three ligands bind more strongly to the C-PC hexamer than the trimer, with the binding sites located at the interface of two (αβ)_3_ trimers. UV/VIS absorption spectroscopy demonstrated the changes in the C-PC absorption spectra in a complex with quercetin and resveratrol compared to the spectra of free protein and ligands. Selected ligands did not affect the secondary structure content, but they induced changes in the tertiary protein structure in the CD study. A fluorescence-based thermal stability assay demonstrated quercetin and coenzyme Q_10_ increased the C-PC melting point by nearly 5 °C. Our study identified food-derived ligands that interact with C-PC and improve its thermal stability, indicating their potential as stabilising agents for C-PC in the food industry.

## 1. Introduction

*Arthrospira platensis*, also known as Spirulina, is a filamentous cyanobacteria whose popularity as an alternative food source is increasing. The annual production of Spirulina, predominantly produced in China, is estimated to be about 10,000 tons [1]. Spirulina’s edible biomass is considered a superfood since it is rich in several bioactive components, including proteins, fatty acids, and micronutrients. Proteins are the most important, and dry Spirulina biomass can contain between 50 and 70% of high-quality proteins [2]. Among these proteins, natural blue pigment C-phycocyanin (C-PC) is the most dominant and crucial bioactive component, whose market value is expected to reach USD 409.8 million by 2030 [3].

C-phycocyanin is an intensively blue-coloured protein due to the presence of a tetrapyrrole prosthetic group called phycocyanobilin (PCB). It is involved in light-harvesting processes in *Arthrospira platensis* as a phycobilisome component. C-PC consists of α (18 kDa) and β (20 kDa) subunits, which form the αβ dimer. The obtained dimer further oligomerises to trimers (αβ)_3_ and hexamers (αβ)_6_ [4]. A PCB chromophore is covalently bound to the protein part via a thioether bond at Cys84 in the α-subunit and Cys82 and Cys153 in the β-subunit [5]. Several studies confirm PCB is responsible for many beneficial effects connected to C-PC. They include immunomodulatory, anti-inflammatory, anti-oxidative, anti-cancerogenic, neuroprotective, and antiviral activity [6,7,8,9]. During digestion or optimised conditions of C-PC proteolysis, bioactive chromopeptides containing PCB could be released [10,11].

C-phycocyanin has an intense blue colour that is rare, making it very interesting for the food and confectionery industry as a natural food colourant. Additionally, C-PC application stretches to biotechnology, medicine and diagnostics [12]. A vital drawback to the broader utilisation of C-PC on an industrial scale is its low stability during food processing since C-PC from Spirulina is sensitive to high temperatures, light and pH. In solution, C-PC’s melting point (T_m_) is about 50 °C; above it, denaturation occurs [13]. Additionally, C-PC is unstable at pH values below 5 and above 8 [14]. Optimal storage conditions for this protein are in the dark at pH 5.5–6.0 and below 45 °C [15]. Several approaches are proposed for increasing the stability of C-PC and phycobiliproteins in general. They include different encapsulation techniques, the addition of edible oils, sugars, whey proteins, cross-linkers, etc. [16,17,18,19]. However, these molecules could stabilise C-PC only at high concentrations (at least in the mM range). On the other hand, the literature data about the specific binding of food-derived ligands to C-PC are scarce. Therefore, identifying high-affinity ligands that could stabilise protein at a much lower ligand concentration (µM range) is a challenge.

**Figure 1:**
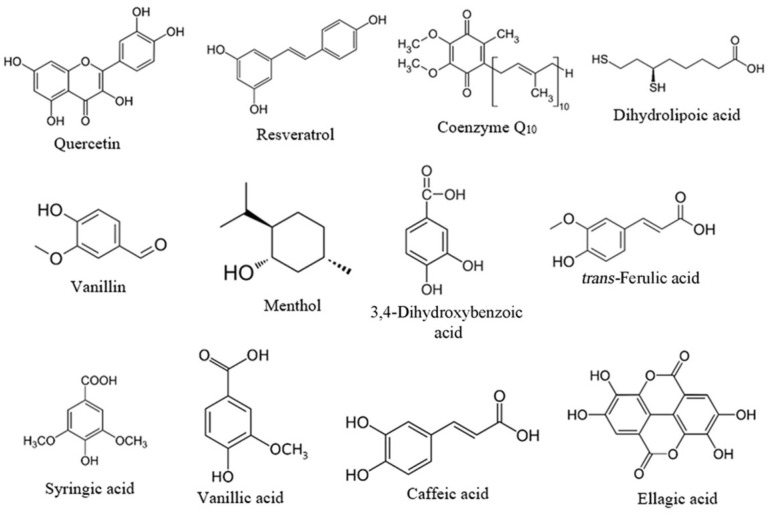
Structures of food-derived ligands used in this study.

This work aims to screen twelve food-derived antioxidants (Figure 1) for their ability to bind to C-PC with high/moderate binding affinities (*K*_d_ at least in the micromolar range) and investigate the consequences of their interactions on the structure and stability of C-PC. Binding, structural investigation and stability were assessed using several spectroscopic techniques, including spectrofluorimetry, UV/VIS absorption spectroscopy and circular dichroism (CD) spectroscopy. Besides the thermal stabilisation of the C-PC, these results also identify molecules for which C-PC could act as a carrier, thus improving its potential bioactivity even further.

## 2. Results

### 2.1. Screening of Suitable Ligands and Calculation of Affinity Constants to C-PC

**Figure 2:**
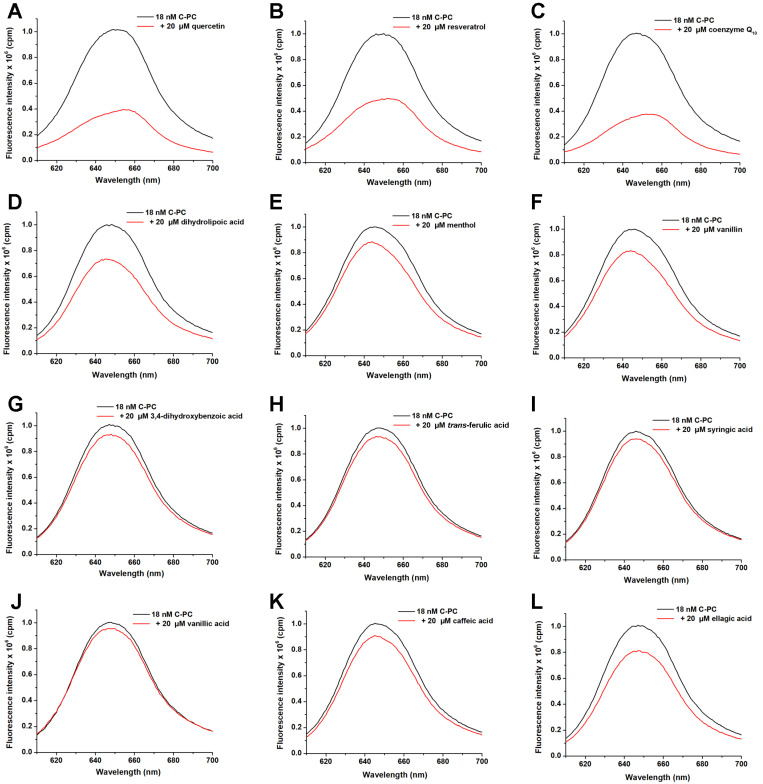
Emission quenching of C-PC in the presence of examined ligands (excitation at 590 nm) at pH 7.2 and 25 °C. Quercetin (**A**), resveratrol (**B**), coenzyme Q_10_ (**C**), dihydrolipoic acid (**D**), menthol (**E**), vanillin (**F**), 3,4-dihydroxybenzoic acid (**G**), *trans*-ferulic acid (**H**), syringic acid (**I**), vanillic acid (**J**), caffeic acid (**K**) and ellagic acid (**L**).

C-phycocyanin exhibits strong fluorescence when it is excited at 590 nm, which enables the study of the interactions of various molecules with this chromoprotein. The addition of ligands caused the fluorescence quenching of C-PC chromophores, indicating that they were binding to C-PC protein (Figure 2).

**Figure 3:**
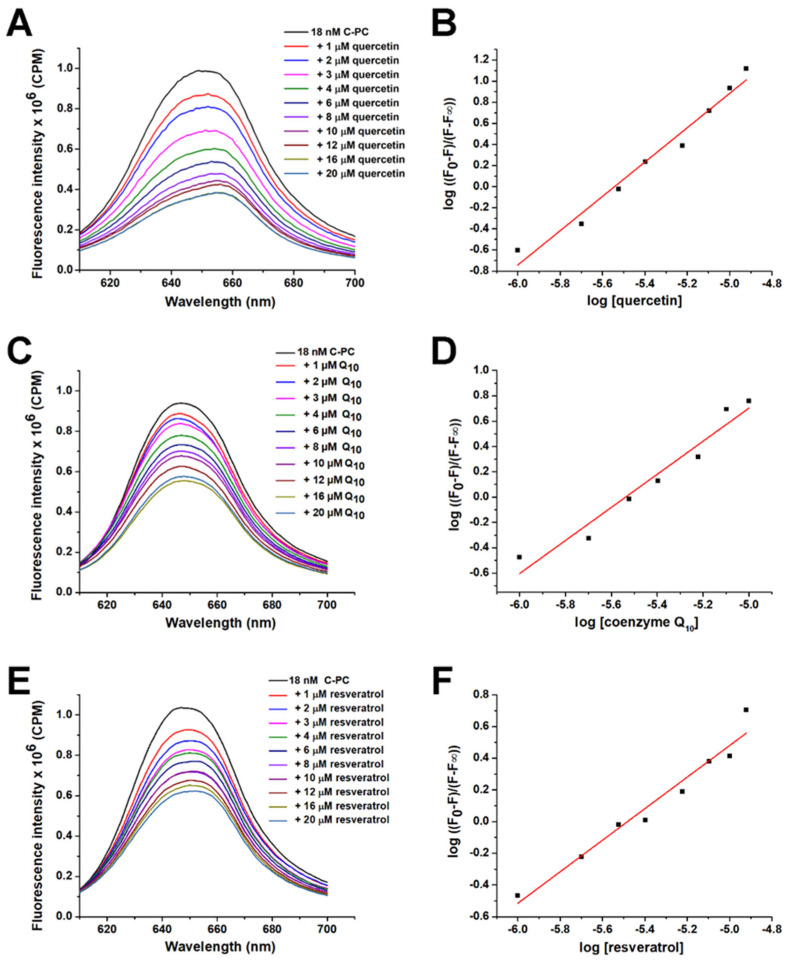
Titration of C-PC with increasing concentrations of quercetin (**A**), coenzyme Q_10_ (**C**) and resveratrol (**E**). Calculation of affinity constants was carried out from Equation (2), based on the corresponding fluorescence quenching data (**B**,**D**,**F**).

Out of 12 ligands tested, only 3 quenched the intrinsic fluorescence of C-PC by more than 30%, indicating decent binding to the protein partner. They included quercetin, resveratrol and coenzyme Q_10_ (Figure 2A, Figure 2B, and Figure 2C, respectively). These three ligands were further analysed, and their binding affinities for C-PC were calculated from the obtained emission spectra (Figure 3; Table 1).

The affinity constants show that quercetin, coenzyme Q_10_, and resveratrol have similar affinities for C-PC, with quercetin having the highest. The binding of these ligands affects the emission maximum of C-PC, suggesting that there are changes in polarity around PCB chromophores. In the presence of coenzyme Q_10_, a slight blue shift of PCB chromophore emission maximum occurs (decreased polarity), while in the presence of resveratrol and quercetin, the redshift occurs (increased polarity).

### 2.2. Molecular Docking Analysis

In this study, we performed molecular docking simulations to determine the binding mode of three ligands (quercetin, resveratrol and coenzyme Q_10_) to C-PC trimers and hexamers, considering the equilibrium between these two forms in aqueous solution [16].

**Figure 4:**
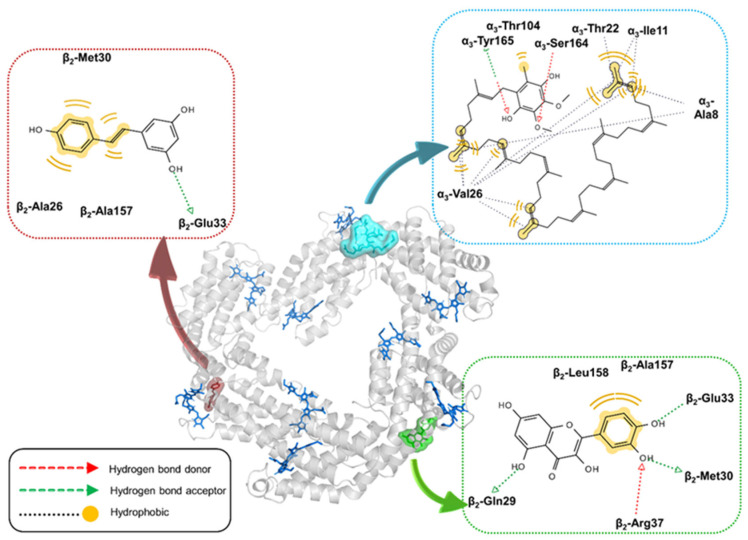
Binding modes of quercetin (green), resveratrol (wine red) and coenzyme Q_10_ (light blue) into C-PC trimer predicted by molecular docking. The molecular surfaces of three ligands are depicted. PCB chromophores are displayed as dark blue sticks. The corresponding ligand interaction diagram (LID) for each ligand is highlighted. The protein structure is visualised in PyMol, and LIDs are calculated in LigandScout 4.4.

The lowest-energy complex formed between a C-PC trimer (αβ)_3_ and quercetin is stabilised through the hydrogen bond interactions, where the hydroxyl group from quercetin’s A-ring interacts with β2-Gln29, and hydroxyl groups from the B-ring interact with β2-Met30, β2-Glu33 and β2-Arg37. Moreover, the B-ring forms hydrophobic interactions with β2-Ala157 and β2-Leu158 (Figure 4). The binding affinities range from −7.6 to −6.6 kcal/mol. Resveratrol also exhibits a preference for the β-subunit, forming hydrogen bonds with β1-Glu33, as well as hydrophobic interactions with β1-Ala26, β1-Met30 and β1-Ala157. The calculated binding affinities for 20 docking poses span from −6.3 to −5.9 kcal/mol (Figure 4). Coenzyme Q_10_, on the other hand, favours the α3-subunit, with the protein–ligand complex stabilised through hydrogen bonding interactions with α3-Ser164 and α3-Tyr165, along with a network of hydrophobic interactions involving the isoprenyl chain with α3-Ala8, α3-Ile11, α3-Thr22 and α3-Val26, as well as the methyl group of the aromatic ring with α3-Thr104. Despite these interactions, its binding affinities are relatively low, ranging from −5.2 to −4.6 kcal/mol (Figure 4).

**Figure 5:**
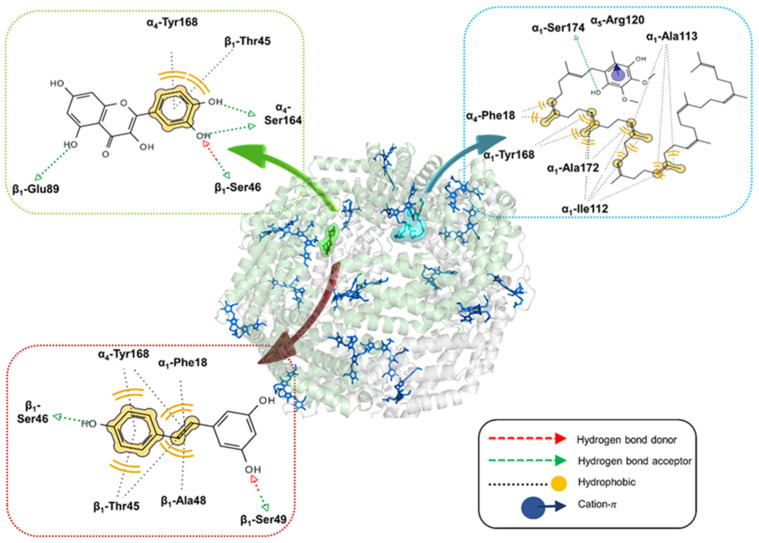
Binding modes of quercetin (green), resveratrol (wine red) and coenzyme Q_10_ (light blue) into C-PC hexamer predicted by molecular docking. Molecular surfaces of quercetin and coenzyme Q_10_ are shown, while resveratrol is represented as wine-red sticks. Two (αβ)_3_ trimers are depicted as gray and pale green ribbons. PCB chromophores are displayed as dark blue sticks. The corresponding ligand interaction diagram (LID) for each ligand is highlighted. The protein structure is visualised in PyMol, and LIDs are calculated in LigandScout 4.4.

For the C-PC hexamer (αβ)_6_, the lowest-energy complex with quercetin is stabilised by a network of non-covalent interactions, including hydrophobic interactions of the B-ring with β1-Thr45 and α4-Tyr168, as well as hydrogen bonding with β1-Ser46, β1-Glu89, and α4-Ser164 (Figure 5). The corresponding binding affinities range from −9.2 to −8.0 kcal/mol. Resveratrol occupies the same binding pocket as quercetin, forming hydrophobic interactions with β1-Thr45, β1-Ala48, α1-Phe18 and α4-Tyr168. Moreover, it establishes hydrogen bonds with β1-Ser46 and β1-Ser49, with binding affinities spanning from −8.1 to −7.6 kcal/mol (Figure 5). On the other hand, coenzyme Q_10_ preferably binds to α-subunits, forming numerous hydrophobic interactions with α1-Ile112, α1-Ala113, α1-Tyr168, α1-Ala172 and α4-Phe18. Furthermore, this ligand is hydrogen-bonded to α1-Ser174 and also forms a cation-pi interaction with the protonated side-chain of α5-Arg120. The calculated binding affinities are the lowest in this series, ranging from −6.4 to −5.9 kcal/mol (Figure 5).

### 2.3. UV-VIS Spectroscopy Analysis

**Figure 6:**
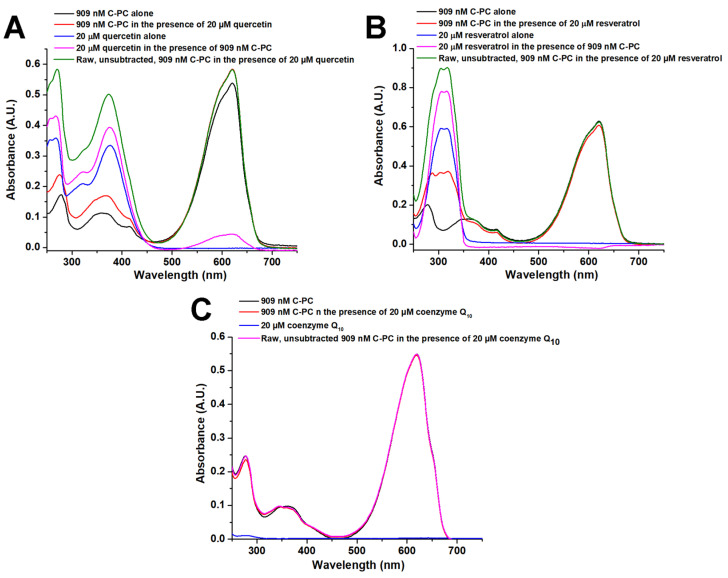
UV-VIS spectra of C-PC and quercetin (**A**), resveratrol (**B**) and coenzyme Q_10_ (**C**) recorded at pH 7.2 and 25 °C. Black and blue lines represent the spectra of free protein and ligands, respectively. Red and pink lines (**A**,**B**) represent differential spectra of C-PC (the spectrum of the ligand is subtracted from the spectrum of the C-PC/ligand complex) and ligands (the spectrum of C-PC alone is subtracted from the spectrum of the C-PC/ligand complex), respectively. Green (**A**,**B**) and pink (**C**) lines represent unsubtracted spectra of protein/ligand complexes.

UV-VIS spectroscopy shows that the presence of quercetin increases the absorbance of the red band (around 620 nm) in the differential spectrum of C-PC (the spectrum of quercetin is subtracted from the spectrum of C-PC: quercetin complex; Figure 6A). The signal of C-PC at 362 nm is also increased, followed by the redshift to 368 nm in the presence of quercetin, thus approaching the wavelength of the absorption maximum of free quercetin (375 nm; Figure 6A). The differential spectrum of quercetin (the spectrum of C-PC alone is subtracted from the spectrum of the C-PC/quercetin complex) also has stronger absorbance compared to free quercetin, and there is no shift in the absorption maximum in the differential spectrum of quercetin compared to the spectrum of free ligands (Figure 6A). Considering these observations, an increase in absorbance in the near-UV range of the C-PC/quercetin complex arises mainly from the higher absorption of quercetin when it is bound to C-PC. A similar effect also occurs with C-PC and resveratrol (Figure 6B). Here, the red band of C-PC is not altered significantly, but the differential spectrum of resveratrol (the spectrum of C-PC alone is subtracted from the spectrum of the C-PC/resveratrol complex) revealed that the absorbance of resveratrol is increased due to complex formation with C-PC. Additionally, the differential spectrum of C-PC in the presence of resveratrol (the spectrum of resveratrol is subtracted from the spectrum of the C-PC/resveratrol complex) exhibits a peak at 320 nm, which corresponds to the position of the resveratrol peak, confirming that interaction between these two molecules induces an increase in resveratrol absorbance. Furthermore, in the same spectrum, an absorbance increase at 280 nm was observed, indicating that aromatic side chains of C-PC are involved in the interactions with resveratrol (Figure 6B). On the other hand, coenzyme Q_10_ does not influence the absorption peaks of C-PC (Figure 6C).

### 2.4. Structural Changes to C-PC Due to Binding of Ligands

**Figure 7:**
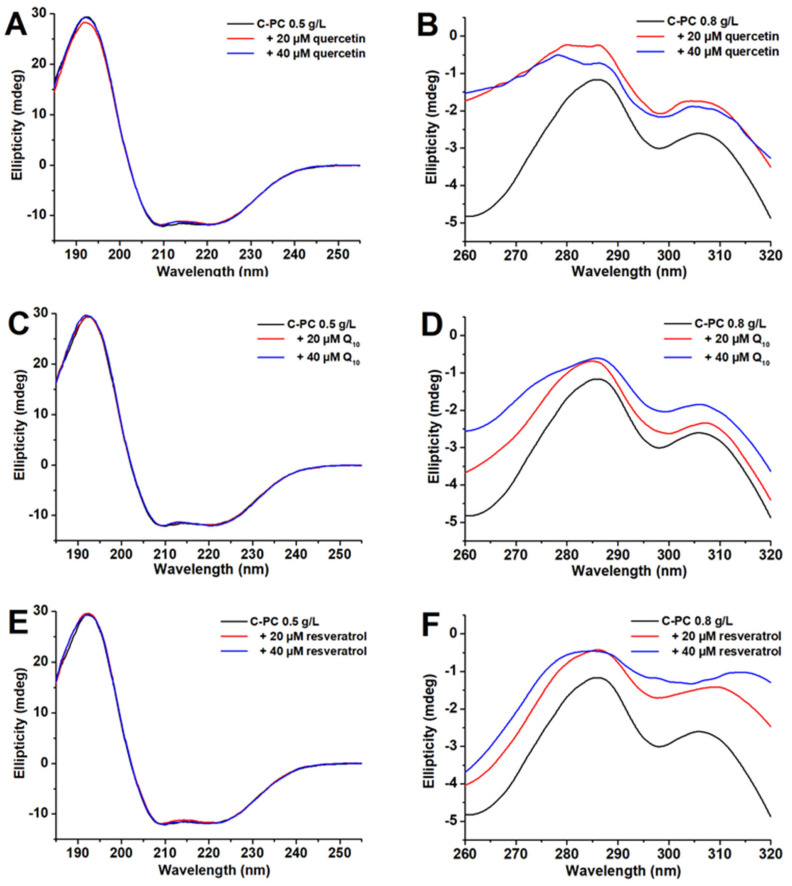
Far-UV (**A**,**C**,**E**) and near-UV (**B**,**D**,**F**) spectra of C-PC in the presence and the absence of quercetin, coenzyme Q_10_ and resveratrol at pH 7.2 and 25 °C.

Far-UV CD spectra (Figure 7A,C,E) of the C-PC solution have two characteristic minima at 209 and 222 nm, confirming that α-helix is the dominant secondary structure, in accordance with the crystal structure of C-PC [20]. Neither ligand significantly influences the far-UV CD spectra (Figure 7A,C,E), suggesting the C-PC secondary structure content in the presence of quercetin, coenzyme Q_10_ and resveratrol is not altered. On the other hand, near-UV CD spectra of C-PC are much more sensitive to ligand binding, confirming the changes in protein tertiary structure upon ligand binding (Figure 7B,D,F). The near-UV CD spectrum of C-PC has two characteristic peaks at 285 and 305 nm. The first peak could arise from Tyr and Trp residues, while the peak at 305 nm arises solely from the Trp residue. However, one αβ dimer of C-PC has 16 Tyr and 1 Trp residues, suggesting that Tyr residues are the major contributor to the band’s intensity at 285 nm. The ligands without C-PC do not exhibit circular dichroism in the near-UV region. Quercetin binding to C-PC induces significant changes in the band shape at 285 nm, followed by the appearance of the additional band at 279 nm (Figure 7B). On the other hand, the binding of coenzyme Q_10_ has a much lower influence on the shape of the protein near-UV CD bands, and the band’s broadening at 285 nm is observed upon the addition of ligand (Figure 7D). Resveratrol at a 20 µM concentration subtly affects the shape of the C-PC near-UV CD bands. In contrast, 40 µM of resveratrol induces the band’s broadening at 285 nm and 2 nm blue shift, while the band at 305 nm is substantially redshifted to 314 nm (Figure 7F).

### 2.5. The Influence of Ligand Binding on Thermal Stability of C-PC

**Figure 8:**
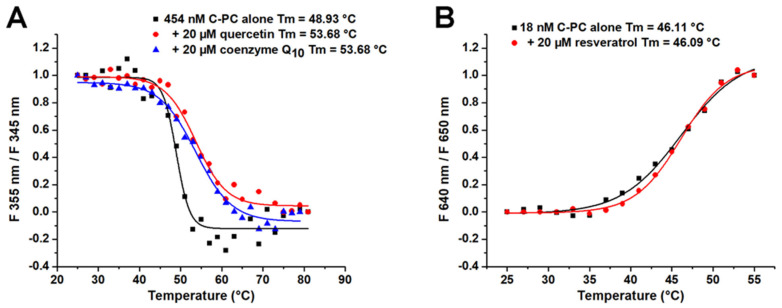
Thermal stability analysis of C-PC alone and in the presence of 20 µM of ligands, including (**A**) quercetin and coenzyme Q_10_ (excitation at 280 nm) and (**B**) resveratrol (excitation at 590 nm) at pH 7.2.

The thermal stability analysis of C-PC alone and in the presence of the tested ligands is presented in Figure 8**.** The effects of quercetin and coenzyme Q_10_ on the thermal stability of C-PC were studied by monitoring the ratio of protein fluorescence at 335 and 345 nm (excitation at 280 nm). The obtained melting curves show that both quercetin and coenzyme Q_10_ improve the thermal stability of C-PC by increasing T_m_ by nearly 5 °C (Figure 8A). On the other hand, resveratrol does not change the melting point of C-PC (Figure 8B). It should be noted that a different approach was used to determine T_m_ for resveratrol. Here, the emission of the C-PC chromophore was followed by excitation at 590 nm. When C-PC was excited at 280 nm in the presence of resveratrol, a strong background signal from the ligand appeared, and T_m_ measurements were turned off at this excitation wavelength. This observation is also the reason why the T_m_ of C-PC alone was somewhat different when the protein was excited at 590 nm (46.1 °C), compared to the excitation at 280 nm (48.9 °C).

## 3. Discussion

In this paper, we probed the ability of twelve food-derived antioxidants to bind and stabilise C-PC, the major protein of cyanobacteria *Arthrospira platensis*. The protein fluorescence quenching approach revealed that quercetin, resveratrol and coenzyme Q_10_ bind to C-PC with the highest affinity, and these ligands were selected for further work. A molecular docking approach was used to predict the binding sites and modes for the selected ligands on the C-PC trimer and hexamer. All three ligands induce changes in the protein conformation, while the binding of quercetin and coenzyme Q_10_ induces the thermal stabilisation of C-PC.

The selected ligands bind to C-PC with a moderate affinity from 2.2 to 3.7 × 10^5^ M^−1^ (the dissociation constant is in the micromolar range). Considering that the concentration of quercetin, resveratrol and coenzyme Q_10_ may be above a few µM in food products [21,22,23], the binding of selected ligands to C-PC could occur in natural food systems, demonstrating the relevance of the characterised binding systems.

Molecular docking analyses reveal distinct binding preferences for quercetin and resveratrol toward β-subunits of the C-PC (αβ)_3_ trimer, while coenzyme Q_10_ interacts preferentially with α-subunits. The identified binding sites for all three ligands on the C-PC hexamer are situated at the interface between two (αβ)_3_ trimers, implying a potential role in stabilising the tertiary structure of C-PC. Quercetin demonstrates the highest binding affinity for C-PC trimers and hexamers, aligning with the highest association constants obtained by fluorescence titration. Despite a rich pattern of intermolecular interactions, the relatively low binding affinity of coenzyme Q_10_ may be attributed to its highly bent conformation, resulting in a substantial energy penalty for a transition from an extended to a bent conformation.

The UV-VIS absorption spectrum of C-PC is an excellent indicator of the conformational state of the protein. This protein has two characteristic bands at a near-UV area at around 360 nm and the so-called red band at about 620 nm in the visible spectrum area, originating from bound PCB [24]. The shape and the ratio of these two absorption maxima are used as an indicator for the C-PC structure, with native protein having higher absorption at 620 nm, where PCB is in linear conformation and tightly bound to C-PC. In a denatured state, PCB obtains a cyclic structure, and in this case, absorption at 360 nm is higher, while the red band is blue-shifted to about 600 nm [24,25]. Quercetin influences C-PC by increasing red band absorption by a small amount. This finding could result from a slight conformational alteration of the PCB chromophore.

As a consequence of binding to C-PC, the intensity at absorption maxima of both quercetin and resveratrol are increased, while the wavelengths of their absorption maxima are not altered. The absorbance spectrum of quercetin is characterised by two prominent bands called I and II. Band I is associated with the cinnamoyl system (B–C ring), and band II with the benzoyl moiety created by the A–C ring. The nature of these transitions is π-π*. In the presence of C-PC, the absorption of both band I and II of quercetin is increased, similar to what is found in its interaction with SDS surfactant [26]. Since no shifts in absorption maxima are detected, the interaction of quercetin to C-PC does not disturb π-π* excitations in both the A-C and B-C ring systems of quercetin. Human serum albumin, for example, causes a robust bathochromic shift of quercetin band I for about 30 nm [27]. Regarding resveratrol, a similar effect was obtained with its interactions with HSA [28]. The obtained results indicate that due to complex formation, aromatic amino acid side chains responsible for C-PC absorption at 280 nm and resveratrol have altered polarity in their environment, leading to increased absorption intensity.

While neither of the ligands could affect the secondary structures of C-PC, the tertiary structure of protein is influenced by the binding of all three ligands, especially in the presence of quercetin and resveratrol. Quercetin binding to C-PC induces significant changes in the shape of the Tyr band, indicating that binding of this molecule occurs in the vicinity of Tyr residues. A similar phenomenon (blue shift of Tyr band) was observed in the near-UV CD spectra of human serum albumin upon quercetin binding [29]. Indeed, our docking results confirmed that quercetin binds in the vicinity of α4-Tyr168. Resveratrol binding influences both bands in the near-UV CD spectra of C-PC, with the more pronounced effect on the Trp band with the substantial redshift (from 305 to 314 nm). However, it is unlikely that Trp residues could exhibit a maximum intensity above 310 nm. Hence, the obtained shift could not arise solely from Trp residue but probably due to the complex formation between this residue and the resveratrol chromophore. Although free resveratrol does not exhibit circular dichroism, its interaction with the aromatic side chains could induce its optical activity reflected in the CD peak at 314 nm. In fact, the resveratrol absorption maximum (320 nm) is close to the observed CD peak, suggesting the significant contribution of resveratrol to the ellipticity of the C-PC/resveratrol complex. The binding of coenzyme Q10 provokes subtle changes in the Tyr band of C-PC, indicating ligand interactions with the Tyr residues within the protein. The docking results also indicated the existence of hydrogen bonding interactions between the phenolic hydroxyl group and α3-Tyr165 for C-PC trimers and hydrophobic interactions with α1-Tyr168 for hexamers.

Considering that a significant drawback of the broader industrial utilisation of C-PC is its lower stability, research with a focus on the stabilisation of C-PC is gaining attention, with several review papers about this subject already being published [13,15]. C-PC has an intense blue colour originating from its prosthetic group, PCB, which is very hard to find in nature [30]. Due to its attractive appearance, C-PC has excellent potential to be utilised as a food colourant. High temperatures represent standard food processing before consumption, and C-PC loses its structure and blue colour at higher temperatures.

Besides colour, denaturation also affects C-PC’s bioactivity [31]. Therefore, the stabilisation of C-PC has two benefits: the preservation of its attractive colour and bioactivity. Another point to consider is the price for food-grade C-PC, estimated to be USD 130 per g [32]. The improved stability of C-PC could significantly impact the cost of its production. Several reports describe thermostable C-PC isolated from extremophilic algae with Tm above 70 °C [33,34]. While their potential for industrial application is greater than C-PC from Spirulina, C-PC is currently dominantly produced from mesophile *Arthrospira platensis*. Cultivating extremophilic algae on a large scale requires many more resources than growing mesophilic algae.

In the literature, there are several approaches for stabilising C-PC at higher temperatures. Based on several studies, it can be concluded that the optimal temperature for keeping C-PC is below 45 °C [15]. There are not many studies, if any, exploring the binding abilities of C-PC with small bioactive molecules. C-PC was shown to interact with and inhibit the activity of α-synuclein and β-secretase, which are involved in fibril formation associated with Alzheimer’s and Parkinson’s disease [35,36]. By forming interactions with BSA, C-PC can also inhibit the formation of BSA fibrils [37]. This study provides insights into the types of molecules that can bind to this bioactive protein and stabilise its tertiary structure at higher temperatures. Furthermore, C-PC can serve as a carrier for these molecules. This further means enriched C-PC preparations can be made with other bioactive small molecules. It was shown that quercetin and coenzyme Q_10_ increase its T_m_ by nearly 5 °C. C-PC’s thermal stability depends on its purity, with a reagent-grade protein having lower stability than a food-grade one [38]. Enriching crude Spirulina extract with the mentioned antioxidants could improve the thermal stability of C-PC even further.

Molecules that bind to C-PC, including quercetin, resveratrol and coenzyme Q_10_, are popular and widely used food supplements. Antioxidants are usually small organic molecules with poor aqueous solubility [39,40]. Combined with their low stability, this represents significant drawbacks in their efficient usage. When bound to proteins, their solubility and bioavailability can increase, as is the case in studies of resveratrol attached to fibrinogen [41] or β-lactoglobulin [42]. Similarly, coenzyme Q_10_ solubility increases due to interactions with casein hydrolysates [43], while quercetin solubility, stability and bioaccessibility are enhanced in excipient emulsions made of milk proteins [44]. In addition, when bound to proteins, the activity of antioxidants can be extended as the protein itself can protect bound ligands from chemical alterations.

## 4. Materials and Methods

### 4.1. Materials

Commercial Hawaiian Spirulina biomass was purchased from Nutrex (Kailua-Kona, HI, USA). All reagents were of analytical grade and were purchased from Sigma (Darmstadt, Germany). C-PC was purified according to the published procedure [45]. The concentration of C-PC was calculated using the following equation [46]:

C-PC _(**mg**/**mL**)_ = (A_615_ − 0.474 × A_652_)/5.34 (1)

where A_615_ and A_652_ are absorbances of isolated C-PC at 615 and 652 nm. The molar concentration of C-PC was calculated by dividing the protein mass concentration by the molar mass of trimeric C-PC (~110 kDa).

Unless otherwise stated, all experiments were performed at room temperature in 20 mM phosphate buffer pH 7.2.

### 4.2. Screening of Antioxidants Binding to C-PC

The binding potential of 12 food-derived antioxidants, including quercetin, resveratrol, coenzyme Q_10_, dihydrolipoic acid, menthol, vanillin, 3,4-dihydroxybenzoic acid, *trans*-ferulic acid, syringic acid, vanillic acid, caffeic acid and ellagic acid (Figure 1), for C-PC was tested.

The binding was analysed using a FluoroMax spectrofluorimeter (HORIBA Scientific, Japan). To C-PC (18 nM, final concentration), 20 µM of each ligand was added. C-PC alone and in the presence of ligands was excited at 590 nm (specific excitation of PCB), and emission spectra were recorded from 610 to 700 nm. Ligands that quenched the emission of C-PC for more than 30% (at the peak maximum of ca. 645 nm) were chosen for further work.

### 4.3. Calculation of Binding Affinity of Selected Ligands to C-PC

C-PC was titrated with increasing concentrations of selected ligands, including quercetin, resveratrol, and coenzyme Q_10_. Spectra were recorded under the same conditions as described in the previous section. The obtained spectra were corrected by subtracting the used ligands’ emission spectra at appropriate concentrations. Considering that the excitation wavelength used was 590 nm, no inner filter effect existed, and thus, it was unnecessary to include its correction in the obtained emission intensity of C-PC. The affinity constant was determined using the following equation:

(2) $$\log\frac{F_{0}-F}{F-F_{\infty }}=n\;\log\left[L\right]+n\;\log K_{a}$$

where F_0_ is the initial emission intensity of C-PC at 645 nm, F is the emission intensity in the presence of ligands, F_∞_ is the emission of C-PC completely saturated with ligands (20 µM), [L] is the molar concentration of ligands and *K*_a_ is the affinity constant in M^−1^. Additionally, the emission of C-PC alone in the presence of increasing concentrations of DMSO or ethanol was recorded, and this change was used for the correction of F and F_∞_.

### 4.4. Molecular Docking

The 3D coordinates of the C-PC (αβ)_6_ hexamer were retrieved from the Protein Data Bank (PDB) with the code 1HA7 [20]. To prepare the protein for docking, we removed all water molecules and adjusted the ionisation state to mimic pH 7.4 using PROPKA [47]. All PCB chromophores were retained in the structure of the macromolecular target. Since the (αβ)_6_ hexamer consists of two stacked trimers, the structure of C-PC (αβ)_3_ was prepared by deleting half of the hexamer structure.

The initial structures of quercetin, resveratrol and coenzyme Q_10_ were downloaded from PubChem, and their starting geometries were generated using the MMFF94s force field [48]. These structures were further optimised using the semi-empirical PM7 method [49] in MOPAC2016 [50], considering the solvation effects of water by applying the COSMO implicit solvation model (EPS = 78.4 keyword).

Molecular docking calculations were performed in AutoDock Vina 1.2. [51]. We expanded the search space to 114 × 112 × 90 Å^3^ to encompass the entire (αβ)_6_ hexamer structure. In the case of the trimer, the search box was set to 114 × 111 × 70 Å^3^. The. pdbqt files of the C-PC protein and three ligands were prepared by removing the apolar hydrogens and assigning default partial atomic charges using the built-in script in Vega ZZ 3.2.3 software [52]. The exhaustiveness was set to 1000, and 20 binding modes were calculated for each ligand. The ligand interaction diagrams were visualised in LigandScout 4.4.3. [53].

### 4.5. UV-VIS Absorption Spectroscopy

Absorbance spectra were recorded on an LLG uniSPEC-4 spectrophotometer (LLG, Meckenheim, Germany) using 909 nM (0.1 mg/mL) C-PC alone and with 20 µM of selected ligands. Spectra were recorded in the range from 250 to 750 nm. For protein and ligands alone, spectra were corrected by subtracting appropriate buffers. For protein and ligands combined, spectra were corrected by subtracting spectra obtained for ligands alone or C-PC.

### 4.6. Circular Dichroism (CD) Spectroscopy

CD spectra were recorded on the J-815 spectropolarimeter (JASCO, Tokyo, Japan). Far UV CD spectra were recorded from 180 to 260 nm using a cuvette with an optical path length of 0.01 cm and protein concentration of 4.54 µM (0.5 mg/mL). Near-UV CD spectra were recorded from 260 to 320 nm using the cuvette with an optical path length of 1 cm and a protein concentration of 9.09 µM (0.8 mg/mL). Spectra were recorded with 3 accumulations and a 50 nm/min scanning speed. Spectra of C-PC were corrected by the subtraction of spectra obtained from ligands alone.

### 4.7. Thermal Stability

The influence of ligands on the C-PC thermal stability was evaluated using fluorescence spectroscopy. C-PC alone (0.05 mg/mL, final concentration) and in the presence of ligands (20 µM, final concentration) was gradually heated from 25 to 80 °C with an increasing rate of 2 °C/min and equilibration time of 1 min. C-PC was excited at 280 nm except for resveratrol, and emission spectra were recorded from 290 to 450 nm. The emissions ratios at 345 and 335 nm were used to construct melting curves. Considering that resveratrol fluorescence interferes with the emission of C-PC in the UV range, its effects on protein thermal stability were studied by C-PC excitation at 590 nm. In comparison, emission spectra were recorded from 600 to 700 nm. The emissions ratios at 640 and 650 nm were used for constructing melting curves. The melting point (T_m_) was determined by fitting the obtained data with a sigmoidal function in Origin software v8.5.1 (USA).

## 5. Conclusions

This study probed the potential of various food-derived antioxidants to bind and stabilise C-phycocyanin (C-PC), a major protein in the cyanobacteria *Arthrospira platensis*, also known as Spirulina. The obtained results revealed that quercetin, resveratrol and coenzyme Q_10_ have the highest binding affinities to C-PC, with dissociation constants in the micromolar range. These binding interactions lead to notable changes in the protein’s tertiary structure. Furthermore, the binding of quercetin and coenzyme Q_10_ induced the thermal stabilisation of C-PC by increasing the melting point by nearly 5 °C.

Our work sheds light on the potential benefits of binding bioactive molecules to proteins. Its significance extends to both the stabilisation of C-PC in the food industry and the possible enhancement of C-PC’s bioactivity through interaction with bioactive food-derived ligands. This is particularly crucial as C-PC is sensitive to temperature, light and pH, limiting its industrial utilisation. Preserving C-PC’s attractive blue colour and bioactivity holds promise for the food industry, where C-PC is considered a food colourant and bioactive nutraceutical.

## Figures and Tables

**Table 1:** Calculated affinity constants (*K*_a_) of selected ligands for C-PC at pH 7.2 and 25 °C.

Ligand	*K*_a_ (M^−1^)
Quercetin	3.7 × 10^5^
Coenzyme Q_10_	3.4 × 10^5^
Resveratrol	2.2 × 10^5^

Values represent the mean of three independent experiments (standard deviation was less than 5%).

## Data Availability

Data are contained within the article.

## References

[1] Thevarajah B., Nishshanka G.K.S.H., Premaratne M., Nimarshana P.H.V., Nagarajan D., Chang J.-S., Ariyadasa T.U. (2022). Large-Scale Production of Spirulina-Based Proteins and c-Phycocyanin: A Biorefinery Approach. Biochem. Eng. J..

[2] Michael A., Kyewalyanga M.S., Lugomela C.V. (2019). Biomass and Nutritive Value of Spirulina (*Arthrospira Fusiformis*) Cultivated in a Cost-Effective Medium. Ann. Microbiol..

[3] Deshmukh R. Phycocyanin Market Expected to Reach $409.8 Million by 2030-Allied Market Research. https://www.alliedmarketresearch.com/press-release/phycocyanin-market.html.

[4] Safaei M., Maleki H., Soleimanpour H., Norouzy A., Zahiri H.S., Vali H., Noghabi K.A. (2019). Development of a Novel Method for the Purification of C-Phycocyanin Pigment from a Local Cyanobacterial Strain Limnothrix Sp. NS01 and Evaluation of Its Anticancer Properties. Sci. Rep..

[5] Shen G., Schluchter W.M., Bryant D.A. (2008). Biogenesis of Phycobiliproteins. J. Biol. Chem..

[6] Jadaun P., Seniya C., Pal S.K., Kumar S., Kumar P., Nema V., Kulkarni S.S., Mukherjee A. (2022). Elucidation of Antiviral and Antioxidant Potential of C-Phycocyanin against HIV-1 Infection through In Silico and In Vitro Approaches. Antioxidants.

[7] Blas-Valdivia V., Moran-Dorantes D.N., Rojas-Franco P., Franco-Colin M., Mirhosseini N., Davarnejad R., Halajisani A., Tavakoli O., Cano-Europa E. (2022). C-Phycocyanin Prevents Acute Myocardial Infarction-Induced Oxidative Stress, Inflammation and Cardiac Damage. Pharm. Biol..

[8] Romay C., Gonzalez R., Ledon N., Remirez D., Rimbau V. (2003). C-Phycocyanin: A Biliprotein with Antioxidant, Anti-Inflammatory and Neuroprotective Effects. Curr. Protein Pept. Sci..

[9] Zhang X., Fan T., Li S., Guan F., Zhang J., Liu H. (2019). C-Phycocyanin Elicited Antitumor Efficacy via Cell-Cycle Arrest, Apoptosis Induction, and Invasion Inhibition in Esophageal Squamous Cell Carcinoma. J. Recept. Signal Transduct..

[10] Liu R.-Z., Li W.-J., Zhang J.-J., Liu Z.-Y., Li Y., Liu C., Qin S. (2022). The Inhibitory Effect of Phycocyanin Peptide on Pulmonary Fibrosis In Vitro. Mar. Drugs.

[11] Minic S.L., Stanic-Vucinic D., Mihailovic J., Krstic M., Nikolic M.R., Cirkovic Velickovic T. (2016). Digestion by Pepsin Releases Biologically Active Chromopeptides from C-Phycocyanin, a Blue-Colored Biliprotein of Microalga Spirulina. J. Proteom..

[12] Eriksen N.T. (2008). Production of Phycocyanin-a Pigment with Applications in Biology, Biotechnology, Foods and Medicine. Appl. Microbiol. Biotechnol..

[13] Pez Jaeschke D., Rocha Teixeira I., Damasceno Ferreira Marczak L., Domeneghini Mercali G. (2021). Phycocyanin from Spirulina: A Review of Extraction Methods and Stability. Food Res. Int..

[14] Su C.-H., Liu C.-S., Yang P.-C., Syu K.-S., Chiuh C.-C. (2014). Solid–Liquid Extraction of Phycocyanin from Spirulina Platensis: Kinetic Modeling of Influential Factors. Sep. Purif. Technol..

[15] Adjali A., Clarot I., Chen Z., Marchioni E., Boudier A. (2022). Physicochemical Degradation of Phycocyanin and Means to Improve Its Stability: A Short Review. J. Pharm. Anal..

[16] Yuan B., Li Z., Shan H., Dashnyam B., Xu X., McClements D.J., Zhang B., Tan M., Wang Z., Cao C. (2022). A Review of Recent Strategies to Improve the Physical Stability of Phycocyanin. Curr. Res. Food Sci..

[17] Zhang Z., Li Y., Abbaspourrad A. (2020). Improvement of the Colloidal Stability of Phycocyanin in Acidified Conditions Using Whey Protein-Phycocyanin Interactions. Food Hydrocoll..

[18] Pradeep H.N., Nayak C.A. (2019). Enhanced Stability of C-Phycocyanin Colorant by Extrusion Encapsulation. J. Food Sci. Technol..

[19] Martelli G., Folli C., Visai L., Daglia M., Ferrari D. (2014). Thermal Stability Improvement of Blue Colorant C-Phycocyanin from Spirulina Platensis for Food Industry Applications. Process Biochem..

[20] Padyana A.K., Bhat V.B., Madyastha K.M., Rajashankar K.R., Ramakumar S. (2001). Crystal Structure of a Light-Harvesting Protein C-Phycocyanin from Spirulina Platensis. Biochem. Biophys. Res. Commun..

[21] García-Martínez D.J., Arroyo-Hernández M., Posada-Ayala M., Santos C. (2021). The High Content of Quercetin and Catechin in Airen Grape Juice Supports Its Application in Functional Food Production. Foods.

[22] Abril M., Negueruela A., Perez C., Juan T., Estopanan G. (2005). Preliminary Study of Resveratrol Content in Aragón Red and Rosé Wines. Food Chem..

[23] Pravst I., Žmitek K., Žmitek J. (2010). Coenzyme Q10 Contents in Foods and Fortification Strategies. Crit. Rev. Food Sci. Nutr..

[24] Scheer H., Kufer W. (1977). Conformational Studies on C-Phycocyanin from Spirulina Platensis. Z. Naturforschung C.

[25] Arciero D.M., Bryant D.A., Glazer A.N. (1988). In Vitro Attachment of Bilins to Apophycocyanin. I. Specific Covalent Adduct Formation at Cysteinyl Residues Involved in Phycocyanobilin Binding in C-Phycocyanin. J. Biol. Chem..

[26] Liu W., Guo R. (2006). Interaction between Flavonoid, Quercetin and Surfactant Aggregates with Different Charges. J. Colloid Interface Sci..

[27] Zsila F., Bikádi Z., Simonyi M. (2003). Probing the Binding of the Flavonoid, Quercetin to Human Serum Albumin by Circular Dichroism, Electronic Absorption Spectroscopy and Molecular Modelling Methods. Biochem. Pharmacol..

[28] Nair M.S. (2015). Spectroscopic Study on the Interaction of Resveratrol and Pterostilbene with Human Serum Albumin. J. Photochem. Photobiol. B Biol..

[29] Vaneková Z., Hubčík L., Toca-Herrera J.L., Furtműller P.G., Mučaji P., Nagy M. (2020). Analysis of Binding Interactions of Ramipril and Quercetin on Human Serum Albumin: A Novel Method in Affinity Evaluation. Molecules.

[30] Pina F., Basílio N., Parola A.J., Melo M.J., Oliveira J., de Freitas V. (2023). The Triumph of the Blue in Nature and in Anthropocene. Dye. Pigment..

[31] Fukui K., Saito T., Noguchi Y., Kodera Y., Matsushima A., Nishimura H., Inada Y. (2004). Relationship between Color Development and Protein Conformation in the Phycocyanin Molecule. Dye. Pigment..

[32] Manirafasha E., Murwanashyaka T., Ndikubwimana T., Yue Q., Zeng X., Lu Y., Jing K. (2017). Ammonium Chloride: A Novel Effective and Inexpensive Salt Solution for Phycocyanin Extraction from Arthrospira (Spirulina) Platensis. J. Appl. Phycol..

[33] Edwards M.R., Hauer C., Stack R.F., Eisele L.E., MacColl R. (1997). Thermophilic C-Phycocyanin: Effect of Temperature, Monomer Stability, and Structure. Biochim. Biophys. Acta Bioenerg..

[34] Ferraro G., Imbimbo P., Marseglia A., Illiano A., Fontanarosa C., Amoresano A., Olivieri G., Pollio A., Monti D.M., Merlino A. (2020). A Thermophilic C-Phycocyanin with Unprecedented Biophysical and Biochemical Properties. Int. J. Biol. Macromol..

[35] Singh N., Hasan S., Kumar J., Raj I., Pathan A., Parmar A., Shakil S., Gourinath S., Madamwar D. (2014). Crystal Structure and Interaction of Phycocyanin with β-Secretase: A Putative Therapy for Alzheimer’s Disease. CNS Neurol. Disord. Drug Targets.

[36] Liu Y., Jovcevski B., Pukala T.L. (2019). C-Phycocyanin from Spirulina Inhibits α-Synuclein and Amyloid-β Fibril Formation but Not Amorphous Aggregation. J. Nat. Prod..

[37] Luo Y.-C., Jing P. (2020). Molecular Interaction of Protein-Pigment C-Phycocyanin with Bovine Serum Albumin in a Gomphosis Structure Inhibiting Amyloid Formation. Int. J. Mol. Sci..

[38] Faieta M., Neri L., Sacchetti G., Di Michele A., Pittia P. (2020). Role of Saccharides on Thermal Stability of Phycocyanin in Aqueous Solutions. Food Res. Int..

[39] Luca S.V., Macovei I., Bujor A., Miron A., Skalicka-Woźniak K., Aprotosoaie A.C., Trifan A. (2020). Bioactivity of Dietary Polyphenols: The Role of Metabolites. Crit. Rev. Food Sci. Nutr..

[40] Chen W., Zou M., Ma X., Lv R., Ding T., Liu D. (2019). Co-Encapsulation of EGCG and Quercetin in Liposomes for Optimum Antioxidant Activity. J. Food Sci..

[41] Gligorijević N., Radomirović M., Rajković A., Nedić O., Ćirković Veličković T. (2020). Fibrinogen Increases Resveratrol Solubility and Prevents It from Oxidation. Foods.

[42] Liang L., Tajmir-Riahi H.A., Subirade M. (2008). Interaction of β-Lactoglobulin with Resveratrol and Its Biological Implications. Biomacromolecules.

[43] Inada A., Oue T., Yamashita S., Yamasaki M., Oshima T., Matsuyama H. (2019). Development of Highly Water-Dispersible Complexes between Coenzyme Q10 and Protein Hydrolysates. Eur. J. Pharm. Sci..

[44] Chen X., McClements D.J., Zhu Y., Chen Y., Zou L., Liu W., Cheng C., Fu D., Liu C. (2018). Enhancement of the Solubility, Stability and Bioaccessibility of Quercetin Using Protein-Based Excipient Emulsions. Food Res. Int..

[45] Zhang Y.-M., Chen F. (1999). A Simple Method for Efficient Separation and Purification of C-Phycocyanin and Allophycocyanin from Spirulina Platensis. Biotechnol. Tech..

[46] Nikolic M.R., Minic S., Macvanin M., Stanic-Vucinic D., Cirkovic Velickovic T., Jacob-Lopes E., Queiroz M., Zepka L. (2020). Analytical Protocols in Phycobiliproteins Analysis. Pigments from Microalgae Handbook.

[47] Olsson M.H.M., Søndergaard C.R., Rostkowski M., Jensen J.H. (2011). PROPKA3: Consistent Treatment of Internal and Surface Residues in Empirical p K a Predictions. J. Chem. Theory Comput..

[48] Halgren T.A. (1999). MMFF VI. MMFF94s Option for Energy Minimization Studies. J. Comput. Chem..

[49] Stewart J.J.P. (2013). Optimization of Parameters for Semiempirical Methods VI: More Modifications to the NDDO Approximations and Re-Optimization of Parameters. J. Mol. Model..

[50] Stewart J.J.P. (1990). MOPAC: A Semiempirical Molecular Orbital Program. J. Comput. Aided. Mol. Des..

[51] Trott O., Olson A.J. (2010). AutoDock Vina: Improving the Speed and Accuracy of Docking with a New Scoring Function, Efficient Optimization, and Multithreading. J. Comput. Chem..

[52] Pedretti A., Mazzolari A., Gervasoni S., Fumagalli L., Vistoli G. (2021). The VEGA Suite of Programs: An Versatile Platform for Cheminformatics and Drug Design Projects. Bioinformatics.

[53] Wolber G., Langer T. (2005). LigandScout: 3-D Pharmacophores Derived from Protein-Bound Ligands and Their Use as Virtual Screening Filters. J. Chem. Inf. Model..

